# A cross-sectional study of medial longitudinal arch development in children with different BMI

**DOI:** 10.3389/fped.2024.1343162

**Published:** 2024-07-26

**Authors:** Jing Chen, Li Tang, Xiaoxuan Yang, Jing Tang, Qingfeng Cheng, Chen Zhao, Han Zhang

**Affiliations:** ^1^ The First Affiliated Hospital of Chongqing Medical University, Chongqing, China; ^2^ Department of Rehabilitation Medicine, The First Affiliated Hospital of Chongqing Medical University, Chongqing, China; ^3^ Department of Endocrinology, The First Affiliated Hospital of Chongqing Medical University, Chongqing, China; ^4^ Department of Orthopedic Surgery, The First Affiliated Hospital of Chongqing Medical University, Chongqing, China; ^5^ Department of Pediatrics, Chongqing High-tech Zone People's Hospital, Chongqing, China

**Keywords:** body mass index, overweight, arch index, arch volume, arch flexibility

## Abstract

**Introduction:**

The influence of excess weight on the development of children's feet remains a subject of debate. To further elucidate whether this relation occurs, this study compared arch morphology and flexibility in three groups of children: underweight, normal, and overweight.

**Methods:**

In total, 1,532 children (807 boys, 725 girls; age range 7–11 years) participated in the study. The researchers measured the participants’ height and weight, calculated their body mass index, and categorized their weight status as underweight, normal weight, or overweight. A three-dimensional foot model was taken using a three-dimensional plantar scanner under non-weight-bearing and weight-bearing conditions to obtain arch morphometric indices (arch index and arch volume) and arch flexibility index (arch volume index).

**Results:**

Analysis of the data showed that the arch index was higher in overweight children compared to underweight and normal weight children (*p* < 0.05), but the differences in arch volume and arch volume index in overweight children compared to underweight and normal weight children were not statistically significant (*p* > 0.05). All children were divided into flatfoot, normal, and pes cavus groups according to arch index, and the arch volume index was statistically significant (*p* < 0.01).

**Conclusions:**

Overweight was not absolutely associated with arch structure and arch flexibility in children from a three-dimensional perspective. Arch development is a long-term process, and it is not clear whether being overweight has an effect on soft tissue or bone formation. Future studies will focus on the effects of long-term overweight on foot structure and arch flexibility in children.

## Introduction

There has been a global surge in obesity across various age groups (1, 2). Excess weight can lead to a range of adverse consequences, including affecting biomechanical changes in the lower limbs (e.g., alignment and structure of the hip, knee, and foot), which are associated with impaired mobility and reduced levels of physical activity (3, 4). Obesity, especially during childhood development, can lead to more serious consequences. According to the World Health Organization, approximately 10% of school-age children aged 5–17 years are overweight, of whom 3% are obese. Considering that the foot is particularly vulnerable to excess weight gain, and that children and adolescents are an important period of physical development (5), children with overweight or obesity may be at increased risk of musculoskeletal problems such as flatfeet. Therefore, the effect of excess weight on the foot structure of children should be a key concern.

To date, the effects of overweight on foot development in children remain controversial, although many studies have been published on the effects of overweight on foot structure in different age groups. Riddiford-Harland et al. found that the Chippaux-Smirak index was higher in obese children compared to their normal-weight peers, indicating a reduction in longitudinal foot arches (5). The correlation between increased body mass index (BMI), foot pain, and flatfeet were found in children and adolescents (6), as well as in adults (7). Mauch et al. (8) found significant differences in BMI and foot type. However, in contrast to all the above research, Evans (9, 10) did not find a positive correlation between increased body weight and flatfoot. As the arch is an elastic and variable three-dimensional (3D) structure, the two-dimensional metrics used in previous studies do not provide complete information, and these contradictory conclusions should be attributed to the lack of an accurate test method to fully represent its properties. On the other hand, radiographs are commonly used to diagnose foot types in adults, but applying them to children carries radiation risks, and incomplete ossification makes it difficult to obtain actual arch features in children. A more accurate indicator of morphology and flexibility is needed to reflect the relationship between body weight and arch morphology.

The arch volume (Va) formed by the projection of the arch surface onto the supporting surface is a new method to reflect the dynamic changes of medial longitudinal arch (MLA) (11–13). Zhao et al. (14) demonstrated that arch volume offers significant advantages in describing morphological alterations of the arch under varying weight-bearing conditions. Overweight may adversely affect the development of children, yet existing tests are difficult to fully characterize the arch and radiographs are not suitable for children. The three-dimensional index Va can better characterize the development of MLA in children, and thanks to Va, we can also obtain the flexibility of the foot arch. The arch volume index (AVI) can also be calculated from arch volume under the non-weight-bearing and weight-bearing arches (11). AVI can represent not only arch flexibility, but the change in mechanical energy due to arch deformation as well. Previous research indicates arch flexibility may be a meaningful description of the relationship between foot structure and foot function (15). Further understanding of arch volume and morphological changes in children with overweight is important to advance the debate on the effects of overweight on arch structure and to develop appropriate three-dimensional clinical measures. Thus, this study aims to explore the connection between excess weight and three-dimensional morphology and flexibility in children.

In this study, foot parameters, such as the arch index (AI), arch volume, and arch volume index, were compared among three groups of children: underweight, normal weight, and overweight. The primary objective of this research was to examine the association between arch morphology and overweight from a three-dimensional standpoint. In addition, the arch morphology of all children was further categorized into the flatfoot group, normal group, and pes cavus group, allowing for a comparison of the disparities in arch flexibility.

## Methods

### Participants

A total of 1,532 children (807 boys, 725 girls; age range 7–11 years) were included in the study. All children aged 7–11 years who had the ability to cooperate independently with the test were included in the study. Children were excluded from study participation if they had a history of lower limb deformity, trauma, surgery, neuromuscular dysfunction, or inability to stand independently. In total, 107 children were excluded from the study on the basis of these criteria. The sex and age of the children were recorded. Height and weight were measured without shoes, from which BMI was calculated. According to BMI, they were divided into three categories: underweight, normal, and overweight.

### Set-up weight-bearing status

In the non-weight-bearing position, children participating in the trial were placed in a height-adjustable seat with the hip and knee joints held at 90° of flexion and the ankle joints in a neutral position. During measurement in the weight-bearing position, participants stood naturally and kept the following landmarks aligned, including the acromion, hip center, knee center, and lateral malleolus.

### Foot scanning

**Figure 1:**
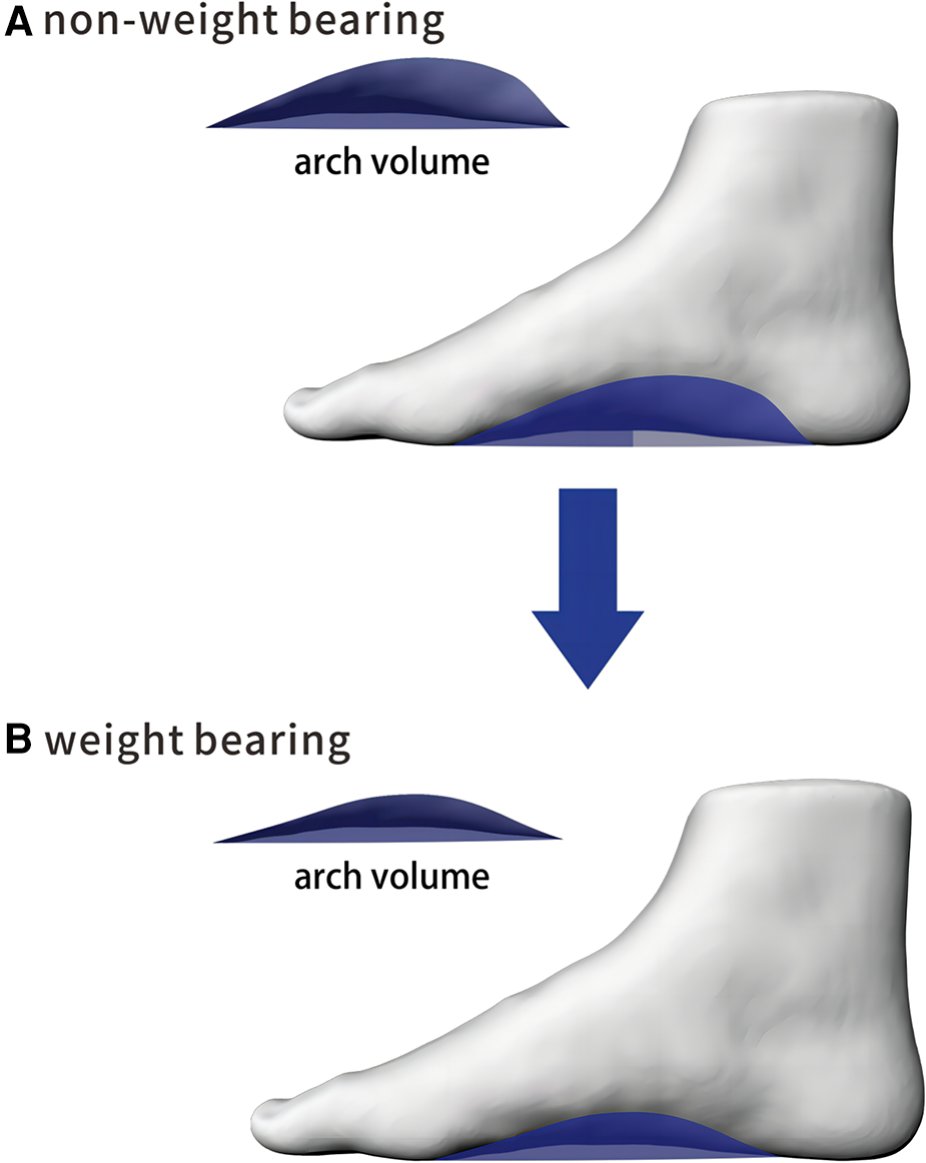
The arch volume: (**A**) non-weight bearing; (**B**) weight bearing.

The “Foot Secret 3D Foot Plantar Scanner” produced by China Shanqi (Chongqing) Wisdom Medical Technology Co., Ltd., was used for 3D foot contour scanning. The scanner used active stereo 3D technology and white light mode projection. Therefore, it is safe for participants’ eyes and can be used in the absence of protective glasses. Referring to the method of Zhao et al. (14), each foot was scanned three times by the “foot Secret 3D Plantar Scanner” in both non-weight-bearing and weight-bearing positions. All scanned feet were placed with the second toe of the foot aligned with the laser axis of the platform. A three-dimensional model of the foot was obtained after scanning, and the AI and Va were obtained from the three-dimensional model. The AI calculated the ratio of the area of the middle third of the footprint to the entire footprint area (16). The Va represents that the arch plane projecting to the supporting surface then formed the volume of the foot arch (11) (Figure 1). The method proposed by Chang et al. (12) was used to calculate the AVI based on the difference in arch volume between non-weight-bearing and weight-bearing conditions, which was calculated as $\mathrm{AVI}=\frac{Va_{\mathrm{non}\text{-}\mathrm{weightbearing}}-Va_{\mathrm{weightbearing}}}{Va_{\mathrm{non}\text{-}\mathrm{weightbearing}}}$_._

### Statistical analysis

The data obtained with the 3D scanner were analyzed using SPSS Software version 19 for Windows (IBM Corp., Armonk, NY, USA), with a significance level of *p* < 0.05. Descriptive statistics (mean, standard deviation, frequencies) were used to analyze the demographic characteristics of the participants. All data were tested for normality using the Kolmogorov–Smirnov test. Data are presented as mean [standard deviation (SD)] or median [interquartile range (IQR)]. Children were classified according to their weight and arch type, respectively. Differences in characteristics between groups were analyzed using the Kruskal–Wallis test with Bonferroni *post-hoc test*s.

## Results

A total of 1,532 children (807 boys, 725 girls; age range 7–11 years) participated in this study. BMI was calculated using height and weight (BMI = weight/height^2^). Participants were categorized according to the method by Ma et al. (17), a BMI table based on age and sex. The statistical details of participants are given in Table 1. The proportions of children with underweight, normal weight, and overweight in this study were 8.68% (133 children), 69.26% (1,061 children), and 22.06% (338 children), respectively. There was no significant difference in age between boys (*n* = 807) and girls (*n* = 725) (*p* = 0.636).

**Table 1:** Demographic characteristics of the participants.

	*N*	Age (years)	Height (cm)	Weight (kg)	BMI (kg/m^2^)	Percent
All	1,532	9.16 ± 1.40	135.45 ± 9.41	32.37 ± 8.35	17.42 ± 2.97	100
Male	807	9.14 ± 1.40	134.96 ± 8.58	32.86 ± 8.15	17.83 ± 3.04	52.68
Female	725	9.17 ± 1.40	135.99 ± 10.24	31.83 ± 8.55	16.95 ± 2.82	47.32
Underweight	133	9.39 ± 1.31	134.85 ± 8.16	25.25 ± 3.70	13.80 ± 0.61	8.68
Normal	1,061	9.15 ± 1.40	134.64 ± 9.41	30.25 ± 6.00	16.51 ± 1.50	69.26
Overweight	338	9.10 ± 1.41	138.24 ± 9.37	41.84 ± 8.62	21.68 ± 2.64	22.06

Bilateral foot models obtained using three-dimensional scanning imaging were used to evaluate foot type and the calculation of “Arch Index” was consistent with the method introduced by Cavanagh and Rodgers (16). A total of 1,532 children were tested in the study and data for 3,064 feet were obtained. The prevalence of flatfoot, normal, and pes cavus in this study was 39.8% (1,218 feet), 57.1% (1,749 feet), and 3.1% (97 feet), respectively (Table 2).

**Table 2:** Summary of foot type diagnosis.

Foot type	BMI			Total
	Underweight	Normal weight	Overweight	
Flatfoot	77 (2.5%)	824 (26.9%)	317 (10.3%)	1,218 (39.8%)
Normal	168 (5.5%)	1,233 (40.2%)	348 (11.4%)	1,749 (57.1%)
Pes cavus	21 (0.7%)	65 (2.1%)	11 (0.4%)	97 (3.1%)
Total	266 (8.7%)	2,122 (69.2%)	676 (22.1%)	3,064 (100%)

Individual differences in children's developmental stages are large, and to eliminate errors in foot size, participants were divided into six groups based on foot length in the weight-bearing position and then analyzed for differences between groups with different BMI. Comparing foot parameters by weight category grouping, except for the two groups of 180–190 and 190–200, there were significant differences in AI between overweight children and normal weight children in all four groups (200–210, 210–220, 220–230, and 230–240) (*p* < 0.05). The data of participants' feet were measured, and the results from descriptive analysis showed that children with overweight had higher arch index in both non-weight bearing and weight bearing positions than children with normal and underweight (Table 3). Thus, overweight children have higher arch index and flatter arches.

**Table 3:** Comparative results of foot parameters in underweight, normal weight, and overweight children in each foot length group.

Foot length	BMI	*N*	Arch index-NW	Arch index-W	Arch volume-NW	Arch volume-W	Arch volume index
180–190	Underweight	25	0.25 (0.03)	0.29 (0.05)	10,690 (3,518)	6,304 (2,941)	0.40 ± 0.16
	Normal	175	0.25 (0.07)	0.30 (0.07)	10,258 (4,103)	7,125 (3,936)	0.33 ± 0.12
	Overweight	19	0.28 (0.08)	0.31 (0.07)	9,699 (5,471)	7,028 (4,442)	0.32 ± 0.11
190–200	Underweight	52	0.24 (0.06)^b^	0.29 (0.05)^b^	12,683 (5,527)	8,478 (3,501)	0.33 (0.18)
	Normal	392	0.25 (0.06)	0.29 (0.06)	12,157 (4,386)	8,215 (4,610)	0.31 (0.18)
	Overweight	80	0.26 (0.06)^b^	0.30 (0.06)^b^	12,197 (5,186)	8,410 (4,004)	0.30 (0.22)
200–210	Underweight	69	0.22 (0.06)^a,b^	0.25 (0.07)^a,b^	16,079 (4,275)^a,b^	11,043 (4,795)^b^	0.28 (0.20)
	Normal	519	0.24 (0.06)^a,c^	0.28 (0.07)^a,c^	14,328 (5,344)^a^	9,910 (5,020)	0.29 (0.19)
	Overweight	110	0.26 (0.06)^b,c^	0.30 (0.07)^b,c^	13,729 (5,291)^b^	9,336 (5,426)^b^	0.30 (0.20)
210–220	Underweight	83	0.22 (0.06)^a,b^	0.26 (0.04)^a,b^	18,051 (7,530)^a,b^	13,696 (6,252)^a,b^	0.26 (0.17)
	Normal	493	0.25 (0.06)^a,c^	0.28 (0.07)^a,c^	16,055 (5,886)^a^	11,434 (5,890)^a^	0.28 (0.20)
	Overweight	159	0.26 (0.08)^b,c^	0.29 (0.07)^b,c^	14,949 (7,383)^b^	11,173 (6,796)^b^	0.29 (0.22)
220–230	Underweight	21	0.25 (0.06)	0.28 (0.06)	17,469 (6,795)	13,644 (5,680)	0.26 (0.12)
	Normal	353	0.24 (0.06)^c^	0.26 (0.06)^c^	19,241 (7,778)	14,057 (7,427)	0.27 (0.20)
	Overweight	172	0.25 (0.07)^c^	0.27 (0.08)^c^	18,389 (7,030)	13,210 (7,867)	0.28 (0.23)
230–240	Underweight	16	0.25 (0.05)	0.26 (0.07)	20,117 (6,483)	13,745 (9,383)	0.31 (0.24)
	Normal	190	0.24 (0.05)^c^	0.26 (0.06)^c^	20,896 (7,180)^c^	15,311 (7,258)^c^	0.25 (0.18)
	Overweight	136	0.25 (0.06)^c^	0.28 (0.08)^c^	19,168 (7,197)^c^	13,559 (6,052)^c^	0.30 (0.24)

NW, non-weight-bearing; W, weight-bearing.

Values are expressed as mean ± standard deviation or median (interquartile range). Significant letters (a–c) are used to label the graphs.

^a^ A significant difference between the underweight group and the normal weight group.

^b^ A significant difference between the underweight group and the overweight group.

^c^ A significant difference between the normal weight group and the overweight group.

Although Va showed the same trend as AI, its descriptive analysis showed that overweight children had lower Va in both the non-weight-bearing and weight-bearing positions than both normal weight and underweight children. However, with the exception of the 230–240 group, there was no statistically significant difference between the non-weight-bearing Va and weight-bearing Va of the children with overweight and normal weight in the other five groups (*p* > 0.05). The arch volume results reflect that the arch volume of children with overweight is not necessarily lower than that of children with underweight and normal weight. In addition, the difference in AVI between children with underweight, normal, and overweight was not statistically significant (*p* > 0.05), suggesting that being overweight also does not necessarily result in reduced arch flexibility in children. The numerical details of these tests can be referenced in Table 3.

**Figure 2:**
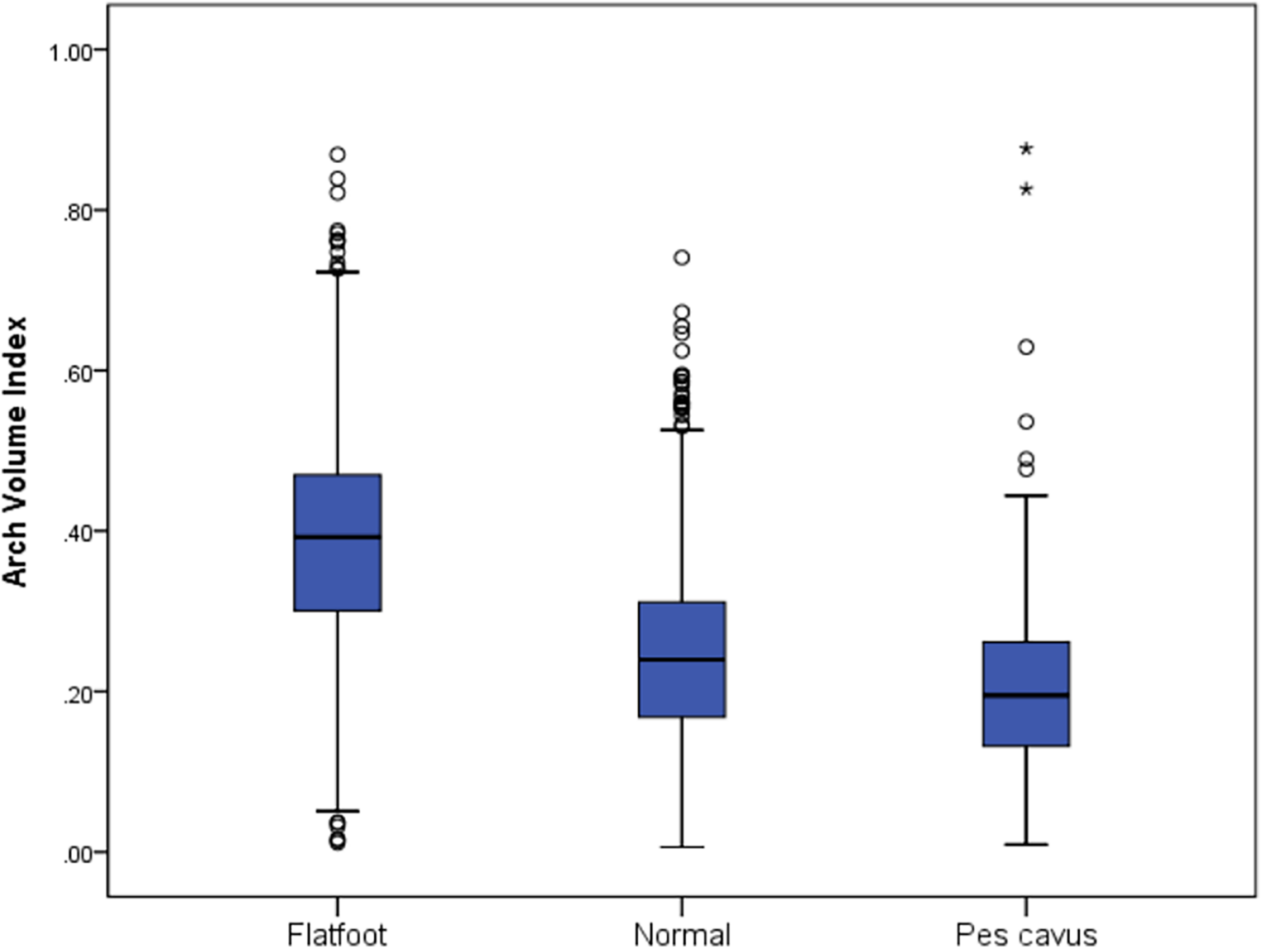
The arch volume index in flatfoot, normal foot and pes cavus.

Further grouping participants by foot type, there were significant differences in AVI among flatfoot, normal foot, and pes cavus (*p* < 0.001). The results of the descriptive analysis showed that the AVI of children with flatfeet was higher than that of children with normal and pes cavus (median = 0.39, median = 0.25, median = 0.22, respectively) (Table 4). The data show that the arch volume index increases sequentially for flatfeet, normal feet, and pes cavus, with flatfeet having higher arch flexibility (Figure 2).

**Table 4:** Comparative results of arch volume index in flatfoot, normal foot, and pes cavus.

	Flatfoot	Normal foot	Pes cavus	H	*p*
Arch volume index	0.39 (0.17)^a,b^	0.24 (0.14)^a,c^	0.20 (0.13)^b,c^	792.19	<0.001

Values are expressed as median (interquartile range). Significant letters (a–c) are used to label the graphs.

^a^ A significant difference between the flatfoot group and the normal group.

^b^ A significant difference between the flatfoot group and the pes cavus group.

^c^ A significant difference between the normal group and the pes cavus group.

## Discussion

The relationship between overweight and foot arch development in children is still controversial because there is no precise testing protocol for foot arches so far due to the individual differences in children's development (18). Following the property that the MLA is a three-dimensional dynamic elastic structure, this study measured, for the first time, foot parameters from two-dimensional (arch index) and three-dimensional perspectives (arch volume) in children aged 7–11 years to determine whether there are differences in the morphology of MLA in overweight children compared to normal weight and underweight children.

The study found that the AI of children with overweight was higher than that of children with normal weight and underweight, and that arch volume showed the same trend of smaller arch volume in children with overweight, which is consistent with previous studies (19, 20). Jankowicz-Szymańska et al. demonstrated that BMI was significantly associated with AI and that excess weight had a greater effect on flatfeet than age (20). Nevertheless, other research presents an ambiguous relationship between excess weight and flatfeet, with Evans failing to identify a positive association between increased body weight and flatfoot (10). On the other hand, the study showed that the prevalence of flatfeet differed from that reported in other studies, with a high prevalence of flatfeet (39.8%) obtained using AI to diagnose foot types. Xu et al. found in a meta-analysis that the detection rate of flatfoot in children in the past 20 years was 25% (21). The prevalence of AI diagnosis of foot type acquisition in children contradicts previous studies, which may be due to the fact that AI is mainly used in adults and the large individual differences in child development may lead to false-positive results in AI-diagnosed foot type. Foot development in children is based on three-dimensional structures, with a focus on the development of morphology and arch flexibility. Va as a three-dimensional index can clearly characterize the developmental trend of foot morphology, and the change of Va in non-weight-bearing and weight-bearing positions can also characterize the arch flexibility of children. The measurement of Va and arch flexibility is useful for further diagnosis and typing of the child's foot.

The relationship between overweight and changes in foot arch morphology was further determined from a three-dimensional perspective. Based on the three-dimensional characteristics of the plantar model, the Va allows visualization of the arch shape and its changes. Changes in arch morphology (i.e., differences in Va) between non-weight-bearing and weight-bearing positions can be represented by AVI. The initial findings showed no significant difference in AVI between underweight, normal, and overweight, suggesting that being overweight does not affect foot flexibility for the time being. Foot types were further classified into flatfoot, normal foot, and high arch. Among these, flatfoot is more flexible, which is consistent with the findings of Zifchock et al. (22). Williams et al. (23) found that different degrees of arch flexibility resulted in different lower limb movement patterns and weight-bearing. A clearer understanding of the relationship between flatfoot and clubfoot can help to develop appropriate clinical treatment plans for foot deformities. It also allows a better description of the foot structure, thus enhancing the predictability of the structure for foot function. Arch flexibility is an important indicator to describe the MLA, but there is no accepted method to classify arch flexibility. AVI can be used as an objective index to evaluate the arch flexibility, which can reflect not only the elasticity of the MLA, but also the mechanical energy change caused by the deformation of MLA. It is valuable to further explore arch morphology and arch flexibility through 3D scanning technology to provide an objective basis for clinical development of interventions.

In addition, although studies have shown no differences in arch volume and arch volume index in children with overweight compared to children with normal weight and underweight, there is a tendency to cause flattening of foot development, especially when children (usually at approximately 6 years of age) begin to engage in activities that place significant demands or loads on the musculoskeletal system of the lower extremities. Stovitz et al. showed that children with overweight/obese and adolescents often report foot pain, which is second only to back pain in prevalence (6). Mickle and Steele also reported that obese adults experience altered foot function and foot pain, which has a direct impact on the patient's mobility and quality of life (24). These findings support the theory that increased pressure on soft tissues and joints, which may be directly related to excess body weight, is associated with an increased incidence of foot discomfort and pain. If overweight lasted for a long time, it may lead to weight gain exceeding the tensile capacity of the plantar fascia, resulting in the formation of flatfoot (25–27). Thus, it is reasonable to hypothesize that the mechanical overload generated by the combination of excess mass and weight-bearing may be detrimental, and more specifically able to affect the structure and function of the developing musculoskeletal structures of the foot. Despite the lack of evidence on the long-term consequences of foot overload, it is noteworthy that children with overweight are less inclined to engage in physical activity (28, 29). Children with excess weight in developmental stages should be given weight control and exercise strategies to prevent the negative consequences of being overweight. It is also noteworthy that follow-up studies are needed to determine the long-term effects of overweight on the MLA. This would allow measurement of the actual evolution of foot structure in children with and without overweight. Finally, it is necessary to provide a multidimensional description of foot structure in childhood. Multidimensional description of foot structure requires a classification of arch height and arch flexibility. It will provide a more reliable basis for clinicians to intervene in overweight and arch collapse.

## Conclusion

From a three-dimensional perspective, overweight is not unequivocally associated with the structure and flexibility of the medial longitudinal arch in children. The arch volume index as a measurement of dynamic changes in arch morphology deserves further study. Arch development is a long-term process, and it is not clear whether being overweight has an effect on soft tissue or bone formation. Future studies will focus on the effects of long-term overweight on foot structure and arch flexibility in children. Clinically, three-dimensional foot parameters can offer orthopedists and pediatricians a more comprehensive understanding of the foot, thereby enabling them to devise preventive measures and interventions with a holistic consideration of the foot.

## Data Availability

The raw data supporting the conclusions of this article will be made available by the authors, without undue reservation.
