# Supplementary figures and images for: A cross-sectional study of medial longitudinal arch development in children with different BMI
Source: Front Pediatr. 2024 Jul 26;12:1343162. doi: 10.3389/fped.2024.1343162 (PMC11310103; doi:10.3389/fped.2024.1343162)

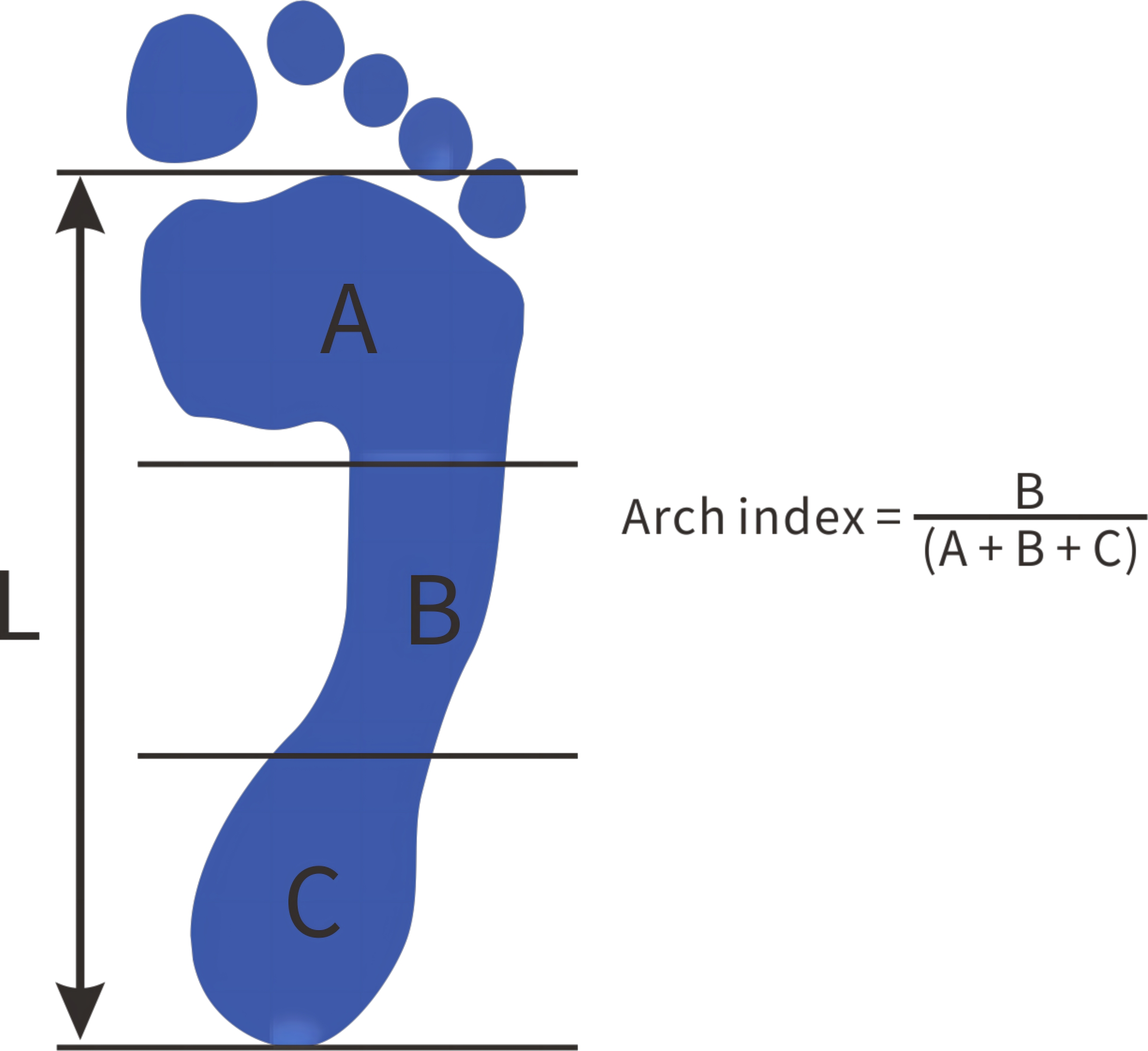

Supplement: Supplementary file 1 [file Image1.jpeg]
